# Predictive factors and clinical efficacy of Chinese medicine Shengji ointment in the treatment of diabetic foot ulcers in the elderly: a prospective study

**DOI:** 10.3389/fphar.2023.1236229

**Published:** 2023-08-16

**Authors:** Yang Zhao, Zheng-Hong Li, Song Sheng, Xin-Yue Dai, Qing-Na Li, Wei-Yi Cao, Rui Gao, Xing-Fang Liu, Hong-Yang Gao

**Affiliations:** ^1^ Xiyuan Hospital China Academy of Chinese Medical Sciences, Beijing, China; ^2^ NMPA Key Laboratory for Clinical Research and Evaluation of Traditional Chinese Medicine, Beijing, China; ^3^ National Clinical Research Center for Chinese Medicine Cardiology, Beijing, China; ^4^ Research Department, Swiss University of Traditional Chinese Medicine, Bad Zurzach, Switzerland

**Keywords:** diabetes, diabetic foot ulcers, Chinese medicine, Shenji ointment, predictive factors, clinical efficacy, treatment outcome, granulation tissue

## Abstract

**Objective:** This study aims to investigate the predictive factors and efficacy of traditional Chinese medicine Shengji Ointment in the treatment of diabetic foot ulcers in the elderly population, with the intent of formulating an effective predictive model for deep diabetic foot ulcer healing. The importance of this research lies in its provision of new perspectives and tools for addressing the severe health impact of diabetic foot ulcers in the elderly population, considering the complexity and diversity of its treatment methods.

**Methods:** The study includes 180 elderly patients with Wagner grade 3-4 diabetic foot ulcers that involve the tendon or fascia. The dependent variable is the initiation time of granulation tissue development. Independent variables encompass demographic information, a treatment strategy including Shengji Ointment, pre-treatment trauma assessment data, routine blood count, and biochemical index test results. Lasso regression is employed for variable selection, and Cox regression is utilized for the construction of a prediction model. A nomogram is generated to authenticate the model.

**Results:** The Chinese Medicine treatment approach, ulcer location, creatinine levels, BMI, and haemoglobin levels are identified as independent predictors of granulation tissue development in diabetic foot ulcers. The combined treatment of Chinese herbal Shengji ointment and bromelain positively influenced granulation tissue development. The location of plantar ulcers, impaired renal functionality, obesity, and anaemia are established as independent risk factors that might influence the speed and probability of ulcer healing. The area under the time-dependent ROC curve fluctuates between 0.7 and 0.8, demonstrating substantial discrimination and calibration of the model.

**Conclusion:** The study ascertains that a combined treatment strategy incorporating Shengji Ointment demonstrates greater effectiveness than the use of cleansing gel debridement alone in facilitating the healing of Wagner grade 3 or higher diabetic foot ulcers. Furthermore, the predictive model developed in this research serves as a valuable tool in evaluating the efficacy of Chinese Medicine treatments like Shengji Ointment for diabetic foot ulcers in the elderly. It aids clinicians in effectively assessing and adjusting treatment strategies, thereby proving its significant application value in clinical practice.

**Clinical Trial Registration:** (https://www.chictr.org.cn/hvshowproject.html?id=73862&v=1.5&u_atoken=b403af53-d3b9-41ae-a7e2-db5498609b0c&u_asession=01tNh69p235bMUO4CmHIXcv8Hxirl5-557Duue9QB5lGfl3mf8IvPlcs2kN2zC30voX0KNBwm7Lovlpxjd_P_q4JsKWYrT3W_NKPr8w6oU7K_AyPrQhedMUWBMR2-ZDL_KO0uwDPR9XlF566xraDvT9mBkFo3NEHBv0PZUm6pbxQU&u_asig=05Kd_Q8fjv-24MVbZpOS9ef3xuCCN-tSVH5eUoJKgNLM7E0-n0zMpW6xLq9gh9aUhkKEEA15rdDoCydncF99APBwVSaTPgEG_V_B1iT4wimdCTxV_4ZVbTlDewxyQtE4YgU4-Oza7KPi94RJ64Utel0yZfqg3Tlm-bVxFNOY-zXFP9JS7q8ZD7Xtz2Ly-b0kmuyAKRFSVJkkdwVUnyHAIJzSYJ6SfhFl0WMTCCasZ7zV2I2qfyrp5m-SELPVeREKgX_6yRmLu26qT8kGfcS-Yaeu3h9VXwMyh6PgyDIVSG1W-7D_Sko5YQtpDbs3uvezYkZcUUY4o9-zDPaoYelmMDs8u7I4TPvtCXaPp44YUJcQ9bHr-_RmKA5V8nji3daArhmWspDxyAEEo4kbsryBKb9Q&u_aref=NNH1nHSUCE6pNvCilV%2F1MD0aERs%3D), identifier (ChiCTR2000039327).

## 1 Introduction

Diabetic foot stands as a serious complication among diabetic patients, thereby constituting a significant public health concern. It correlates with elevated rates of disability, recurrence, protracted hospital stays, complex clinical management, and an unfavorable long-term prognosis (American Diabetes Association, 2018). Among the various presentations of diabetic foot, diabetic foot ulcers (DFUs) are the most common, with three-fourths of diabetic foot patients suffering from severe foot infections. This results in a 10–30 times higher incidence of lower extremity amputations in patients with DFUs compared to the general population (Trautner et al., 1996), contributing to the majority of amputations among diabetic patients—accounting for over 85% of instances (Chinese Society of Medical Sciences et al., 2019a). The Wagner grade is broadly employed to evaluate the classification of DFUs (Trautner et al., 1996). As per a nationwide study, 45% of 669 patients were diagnosed with Wagner grade 3 or higher, with the overall amputation rate nearing 20% (Jiang et al., 2015). Moreover, a gradual decline in wound healing rate was observed with an increasing Wagner grade (Fei et al., 2012). Patients who have undergone amputation of one limb bear a high risk of developing amputation of the contralateral limb. Furthermore, 30%–50% of initial amputations lead to subsequent amputations within 1–3 years. Patients with diabetes mellitus saw the greatest increase in amputation rates, rising from 10 per 100 patients with lower extremity ulcers in 2005 to 28 per 100 patients in 2013 (Humphries et al., 2016). These findings underscore a surging prevalence of DFUs involving deeper tissues, such as the tendon fascia, thereby emphasizing the urgent need for more effective treatments to improve prognosis.

DFUs are traditionally managed through physical debridement, wound repair, wound decompression, and negative pressure therapy. The formation of granulation tissue is recognized as a reliable indicator of wound healing (Lin et al., 2005; Valenzuela-Silva CM et al., 2013). However, the effectiveness of these treatment strategies differs among various types of DFUs. Bacteria adhere to the wound surface, proliferate, establish a biofilm, and gradually impair the normal tissue. Once formed, eradicating the biofilm becomes challenging with conventional treatments, including antibiotics and physical debridement (Sanchez et al., 2013; Kalan et al., 2016). Patients with deep ulcers generally exhibit a poorer prognosis, and over-treatment of deep ulcers that expose tendons could potentially culminate in the loss of limb function (Mohajeri-Tehrani et al., 2016; Chinese Society of Medical Sciences et al., 2019b).

The potential role of Chinese herbal medicine in promoting superficial ulcer healing and mitigating disability has been explored as a prospective treatment strategy for diabetic foot, as supported by previous studies (Guo, 2005; Liu et al., 2021). The combined traditional Chinese medicine treatment involves two types of medicine—Shengji ointment and bromelain, originating from Zhang Tianlei’s “Compendium of Ulcerology.”

Shengji ointment (Chinese patent medicine with China Food and Drug Administration approval number: Z12020345) consists of Angelica sinensis (Oliv.) Diels (Apiaceae; Angelicae sinensis radix), Rehmannia glutinosa (Gaertn.) DC. (Orobanchaceae; Rehmanniae radix), Gypsum Fibrosum [main component: CaSO(4)], Crinis Carbonisatus (known as Xue-yu-tan in Chinese), calamina and beeswax. The names of the plants were verified on http://www.plantsoftheworldonline.org. Shengji ointment (30 g per bottle, lot number: YW202000285) was supplied by Tianjin Darentang Jingwanhong Pharmaceutical Co., Ltd., (Tianjin, China), with an expiration period of 36 months. The drug adheres to the quality standards set by the National Medical Products Administration (NMPA) National Drug Standard Revision Approval (2001ZFB0067) and NMPA Supplementary Drug Application Approval (2017B02035).

Ultra-performance liquid chromatography with quadrupole time-of-flight mass spectrometry instrument (UPLC-QTOF-MS) was used to identify the components of Shengji ointment. The chemical analysis follows the standards established by the ConPhyMP statement (Heinrich et al., 2022). The UPLC fingerprints show that the active ingredients of Shengji ointment include Azelaic acid, N-butylidenephthalide, 3-N-butylphthalide, Palmitoleic acid, Ligustilide, Linolenic acid, Linoleic acid, Palmitic acid, Oleic acid. Detailed information on the botanical drug extraction and formulation process, quality control methods, and UPLC chromatogram of Shengji ointment ingredients can be found in Supplementary Appendix S3.

The pineapple protease (bromelain), sourced from Shantou Olive Pharmaceutical Co., Ltd., (Guangdong, China), has a 24-month expiration period. It is approved by China Food and Drug Administration (approval number: H44024825) and has been widely used as a complementary treatment for Hypercholesteremic, combined hyperlipidemia and cardiovascular disease (CVD) in China. The combined treatment process involves the enzymatic decomposition of the fibrin clot at the inflamed area, sparing the basal sore surface bed from mechanical debridement damage, and establishing a moist healing environment for the sore surface with Shengji ointment.

However, the low quality of existing studies restricts their ability to provide high-quality clinical evidence-based findings. Through this multi-center clinical trial, we sought to determine whether combined Chinese Medicine treatment constituted an independent protective factor for wound healing. Our aim was to establish a practical predictive tool for clinical management by examining the factors associated with the healing of deep DFUs. Simultaneously, we endeavored to devise a clinical prediction model for patients with Wagner grade 3-4 injuries involving the tendon or fascia tissue.

## 2 Materials and methods

### 2.1 Design and participants

Data for this study were derived from a multicenter, open-label, randomized controlled trial. This research assessed the treatment outcomes of 180 patients with diabetic foot ulcers, who were enrolled from four hospitals in Tianjin and Shanxi between 1 January 2021, and 31 December 2021. Adult patients who satisfied the following criteria were included: 1) diagnosed with diabetic foot disease and Wagner grade 3–4 ulcers exposing tendon tissue; 2) fasting blood glucose ≤10 mmol/L; 3) targeted ulcer debridement area ranging between 1 and 20 cm^2^ (for patients with multiple lesions, the largest ulcer was deemed the target lesion); 4) an ankle-brachial index ≥0.5 on the side of the limb harboring the ulcer; 5) voluntary participation and signed informed consent. Patients were excluded from the study if they met any of the following criteria: 1) presence of malignant lesions and severe infection within the ulcer; 2) severe uncontrolled hypertension (systolic blood pressure ≥160 mmHg or diastolic blood pressure ≥110 mmHg); 3) severe malnutrition (serum albumin level <28 g/L or hemoglobin <90 g/L or platelet count <50 × 10^9^/L); 4) allergic disposition or allergies to the constituents of the treatment under investigation and reference drugs; 5) cognitive dysfunction that impeded fully informed consent or an inability to complete the trial or comply with its requirements, according to the researcher’s judgment.

### 2.2 Interventions

In the intervention group, patients received a combined topical treatment regime comprising Chinese herbal Shengji ointment and bromelain. In the control group, a hydrocolloid dressing was applied to cover the wound. Both groups received treatment once every 24 h for a 4-week period, alongside routine medical and surgical treatment (including glycemic control, blood pressure reduction, lipid regulation, antiplatelet medication, debridement, and others). No additional Chinese or Western drugs relevant to ulcer treatment (such as vasodilators including lipid microsphere prostaglandin injections, beraprost sodium, cilostazol, and others; antiplatelet drugs such as aspirin and clopidogrel; anticoagulant drugs such as unfractionated heparin or low-molecular-weight heparin, and oral anticoagulants; or TCM or proprietary Chinese medicine, topical antibiotics, and others, which promote muscle growth and act as astringents in sore treatment) were allowed during the trial. This restriction extended to biological treatments (like stem cell therapy, topical autologous platelet-rich plasma, and TCM or Chinese herbal tonics with functions similar to the trial treatment).

**Table:** 

Treatment	Treatment 1 in the intervention group: Shengji ointment	Treatment 2 in the intervention group: Pineapple protease (bromelain)	Treatment in the control group: Comfeel^®^ plus wound dressing
Size	30 g/bottle	10,000 units	25 g/piece
Formulation	Ointment	Tablet	Hydrocolloid dressing
Usage and dosage	For external use. The ointment will be spread on skimmed cotton and applied to the affected area	For external use. It will be applied to exposed tendons and areas with necrotic tissue	Apply Comfeel^®^ dressing to the ulcer. Ensure the dressing height is at the level of the surrounding skin. Then, apply a layer over the ulcer and surrounding area
Route of administration	Topical	Topical	Topical
Frequency of administration	Once every 24 h	Once every 24 h	Once every 24 h
Treatment course	4 weeks	4 weeks	4 weeks
Manufacturer	Tianjin Darentang Jingwanhong Pharmaceutical Co., Ltd., Tianjin, Tianjin, China	Shantou Olive Pharmaceutical Co., Ltd., Shantou City, Guangdong, China	Coloplast Group, Humblebaek, Denmark

### 2.3 Outcomes

Our primary outcome was the time to granulation, as new granulation is closely linked with ulcer treatment prognosis. Granulation time (in days) was defined as the period from the enrolment day to the emergence of new granulation tissue within the wound. This study was a non-blinded clinical trial; an independent evaluator captured images of the target ulcer. Using 3D scanning, we obtained the wound surface topography. We then utilized the inSight^®^ platform (eKare, Inc., Fairfax, VA, United States) to identify different tissue types and measure the areas they covered. This approach effectively controlled bias in efficacy evaluation arising from the non-blinded methodology. Additionally, we assessed demographic data, medical and allergy history, vital signs, blood routine, C-reactive protein, urine routine, liver and renal function, and wound assessments.

### 2.4 Allocation

We employed the central randomized MagMinDA clinical trial grouping system for participant enrollment. This process ensured the implementation of randomization concealment, effectively averting the introduction of selection bias.

This study received ethical approval from the Medical Ethics Committee of the Second Affiliated Hospital of Tianjin University of Chinese Medicine (Approval number: 2020-006-01). It was also registered with the China Clinical Trials Registry (Registration number: ChiCTR2000039327) on 23 October 2020.

### 2.5 Data capture

This study utilized an Electronic Data Capture (EDC) system to gather patient case information and test examination data. To ensure patient privacy, any personally identifiable information was encoded and processed before data extraction and analysis.

The analysis incorporated 35 variables, encompassing demographic data such as gender, age, marital status, and education level. It also considered physical characteristics such as height, weight, blood glucose levels, and routine pre-treatment blood tests, including red blood cell count, white blood cell count, hemoglobin, and platelet count. Pre-treatment biochemical indicators of liver and kidney function—namely, glutamic transaminase, glutamic oxaloacetic transaminase, total bilirubin, direct bilirubin, indirect bilirubin, total protein, albumin, creatinine, and uric acid—were also assessed.

Furthermore, a detailed analysis was conducted on wound assessment information, such as the maximum ulcer depth, ulcer area, ulcer volume, tissue type (granulation tissue/necrotic/crust), specific wound location, the area of granulation tissue within the ulcer, Wagner classification, and the ankle-brachial blood pressure ratio of the ulcerated limb.

### 2.6 Analysis methods

In this study, data analysis was undertaken using R version 4.1.2. Quantitative metrics were reported as the mean ± standard deviation. The statistical tests employed included the independent *t*-test for variables with a normal distribution and the Wilcoxon rank-sum test for variables with a skewed distribution. Qualitative metrics were expressed in terms of frequencies and percentages and were analyzed using the Chi-square test with a significance level set at α = 0.05.

The modeling group utilized data from centers 1, 3, and 4, while the external validation group used data from center 2. Considering the small sample size and a large number of predictor variables, lasso regression was employed for variable screening in the modeled data. *λ* was selected using 10-fold cross-validation of lasso regression, where the corresponding *λ* was the lasso screening variable criterion in this study when the mean error value was at its minimum.

After variable screening based on lasso regression, Cox regression clinical prediction models were developed, and regression coefficients, standard error values, HRs, 95% confidence intervals, and *p*-values were computed for each variable in the model. Nomograms were also plotted. External validation was conducted using the equation fit data derived from the modeling set. Model evaluation was performed on both modeled and validated data sets. Model discrimination was evaluated using the area under the time-dependent ROC curve (AUC), which is generally considered moderately discriminatory between 0.70–0.80.

## 3 Study results

### 3.1 Baseline analysis

This study enlisted a total of 180 participants, with 126 assigned to the modeling group and 54 to the validation group. A comparative analysis of the demographic and baseline data between the two groups was conducted, and the results are detailed in Table 1.

**TABLE 1:** Demographics and baseline information. Comparison of baseline characteristics in the modeling and validation groups.

Variables	Modelling group	Validation group	*p*
*N*	126	54	
Age (years)	64.64 ± 10.30	62.23 ± 11.20	0.213
Duration of the illness (in months)	9.25 ± 18.13	5.04 ± 8.88	0.075
Height (cm)	168.51 ± 7.81	168.64 ± 7.51	0.923
Body weight (kg)	68.99 ± 11.80	69.66 ± 12.73	0.763
Fasting blood glucose (mmol/L)	6.80 ± 1.64	6.91 ± 2.05	0.725
Postprandial 2H glucose (mmol/L)	10.48 ± 1.68	10.38 ± 2.05	0.772
Red blood cell count (10^12^/L)	4.14 ± 0.57	4.16 ± 0.61	0.819
White Blood Cell Count (10^9^/L)	7.10 ± 3.12	7.49 ± 2.20	0.459
Haemoglobin (g/L)	121.11 ± 20.46	121.67 ± 19.25	0.881
Platelets (10^9^/L)	266.92 ± 82.84	273.18 ± 95.65	0.692
Alanine aminotransferase (ALT) (U/L)	24.36 ± 54.66	18.10 ± 9.97	0.574
Aspartate aminotransferase (AST) (U/L)	20.85 ± 22.84	16.90 ± 6.67	0.7
Total bilirubin (umol/L)	8.96 ± 4.59	7.10 ± 4.43	0.001
Direct bilirubin (umol/L)	2.89 ± 1.88	3.41 ± 1.82	0.069
Indirect bilirubin (umol/L)	6.24 ± 3.88	3.55 ± 2.78	<0.001
Total protein (g/L)	68.38 ± 6.96	67.92 ± 7.14	0.718
Albumin (g/L)	38.51 ± 5.45	38.14 ± 4.06	0.697
Creatinine (umol/L)	74.77 ± 51.25	73.87 ± 30.47	0.811
Uric acid (umol/L)	300.54 ± 97.43	304.26 ± 97.36	0.835
Maximum depth (cm)	0.65 ± 0.74	0.42 ± 0.40	0.102
Ulcer area (cm^2^)	8.70 ± 6.22	6.45 ± 5.78	0.028
Ulcer volume (cm^3^)	3.78 ± 6.52	2.24 ± 3.81	0.051
Percentage of granulation (%)	29.63 ± 24.80	43.59 ± 28.78	0.008
Percentage of decay (%)	43.25 ± 28.78	33.38 ± 32.43	0.038
Percentage of crust (%)	27.27 ± 26.23	23.18 ± 23.12	0.357
Area of granulation tissue within the wound (cm^2^)	2.35 ± 2.63	2.76 ± 3.15	0.486
Total wound area (cm^2^)	8.74 ± 6.25	6.45 ± 5.78	0.026
Ankle brachial index (ABI)	0.78 ± 0.09	0.82 ± 0.10	0.035
Whether granulation occurred			0.107
No	8 (6.35%)	0 (0.00%)	
Yes	118 (93.65%)	54 (100.00%)	
Treatment			0.745
Hydrocolloid dressing	62 (49.21%)	28 (51.85%)	
Shengji ointment and bromelain	64 (50.79%)	26 (48.15%)	
Ulcer location			0.127
Dorsum of foot	26 (20.63%)	17 (31.48%)	
Toe	69 (54.76%)	21 (38.89%)	
Plantar	31 (24.60%)	16 (29.63%)	
Gender			0.645
Male	70 (55.56%)	32 (59.26%)	
Female	56 (44.44%)	22 (40.74%)	
Marital status			0.562
Unmarried	2 (1.59%)	2 (3.7%)	
Married	114 (90.48%)	46 (85.19%)	
Divorced	2 (1.59%)	2 (3.70%)	
Widowed	6 (4.76%)	4 (7.41%)	
Unknown	2 (1.59%)	0 (0.00%)	
Educational level			0.518
Illiterate	2 (1.59%)	0 (0.00%)	
Primary education	16 (12.70%)	7 (12.96%)	
Lower secondary	41 (32.54%)	21 (38.89%)	
High School	24 (19.05%)	8 (14.81%)	
College	5 (3.97%)	4 (7.41%)	
University and above	1 (0.79%)	2 (3.70%)	
Not available	37 (29.37%)	12 (22.22%)	
Wagner Grading			0.086
Grade 3	74 (58.73%)	39 (72.22%)	
Grade 4	52 (41.27%)	15 (27.78%)	

### 3.2 Lasso regression variable screening

**FIGURE 1:**
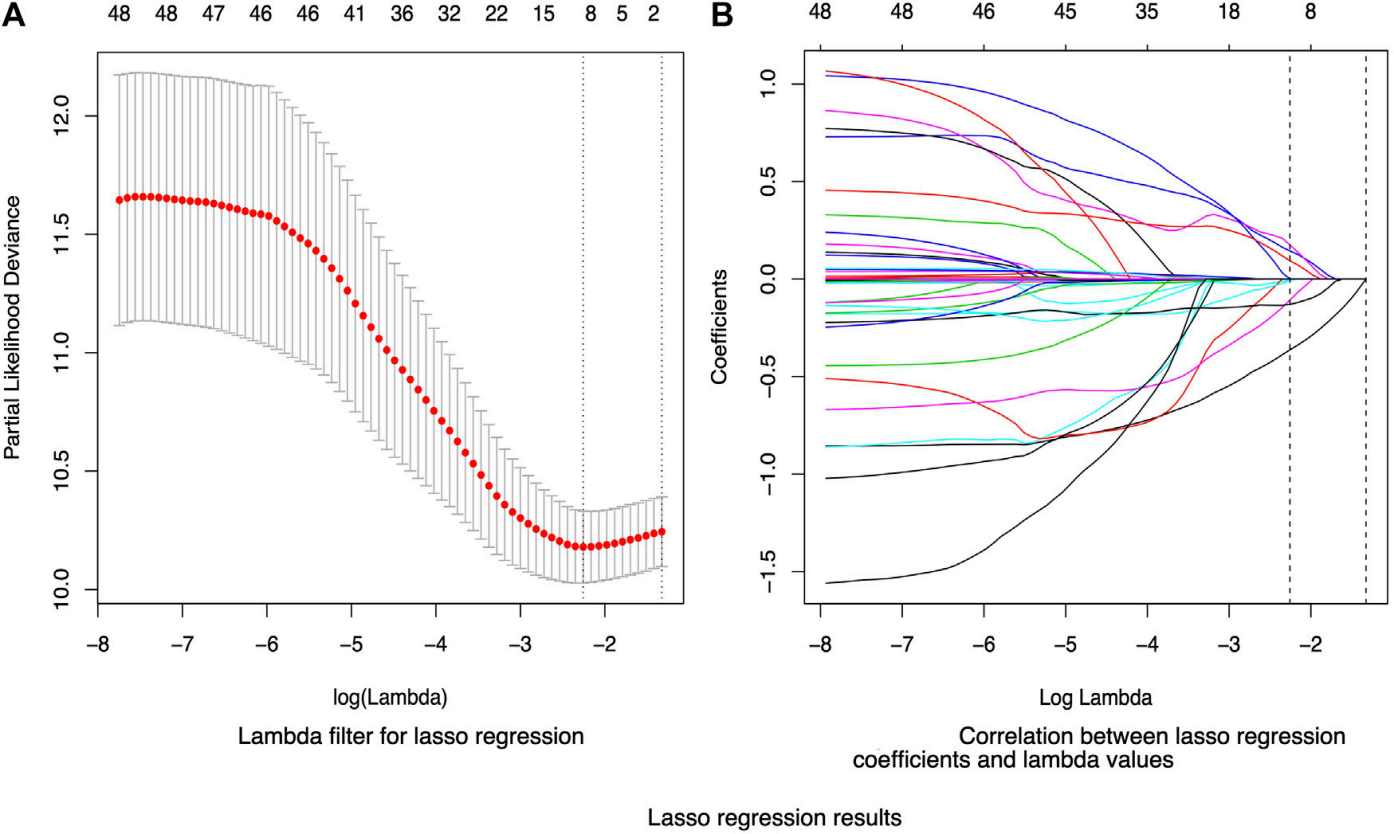
Lasso regression results. **(A)** presents the tuning parameter (lambda) selection in the lasso model using 10-fold cross-validation via the lambda.min and lambda.1se criteria. The *y*-axis represents the partial likelihood deviance, the lower *x*-axis indicates log (λ), and the upper *x*-axis represents the number of variables included in the model. **(B)** showcases the LASSO coefficient profiles of the variables. The *y*-axis represents the value of the coefficient, and the lower *x*-axis denotes log (lambda).

In this research, we assessed 35 potential factors implicated in the formation of granulation tissue. The Lasso method was employed to identify the variables exerting the most significant impact on the outcome. The optimal *λ* parameter in the Lasso model was ascertained through ten-fold cross-validation. The vertical line in Figure 1A represents the optimal value with the smallest *λ* and its standard error (SE). The optimal model value was established to be *λ* = 0.1047 and log(λ) = −2.2566. The Lasso regression process highlighted treatment, ulcer location, hemoglobin, creatinine, the proportion of the decaying area, Body Mass Index (BMI), disease duration, albumin, and the ankle-brachial index as critical variables. The coefficient profiles for all variables are illustrated in Figure 1B.

### 3.3 Construction of the Cox proportional hazard regression model

**FIGURE 2:**
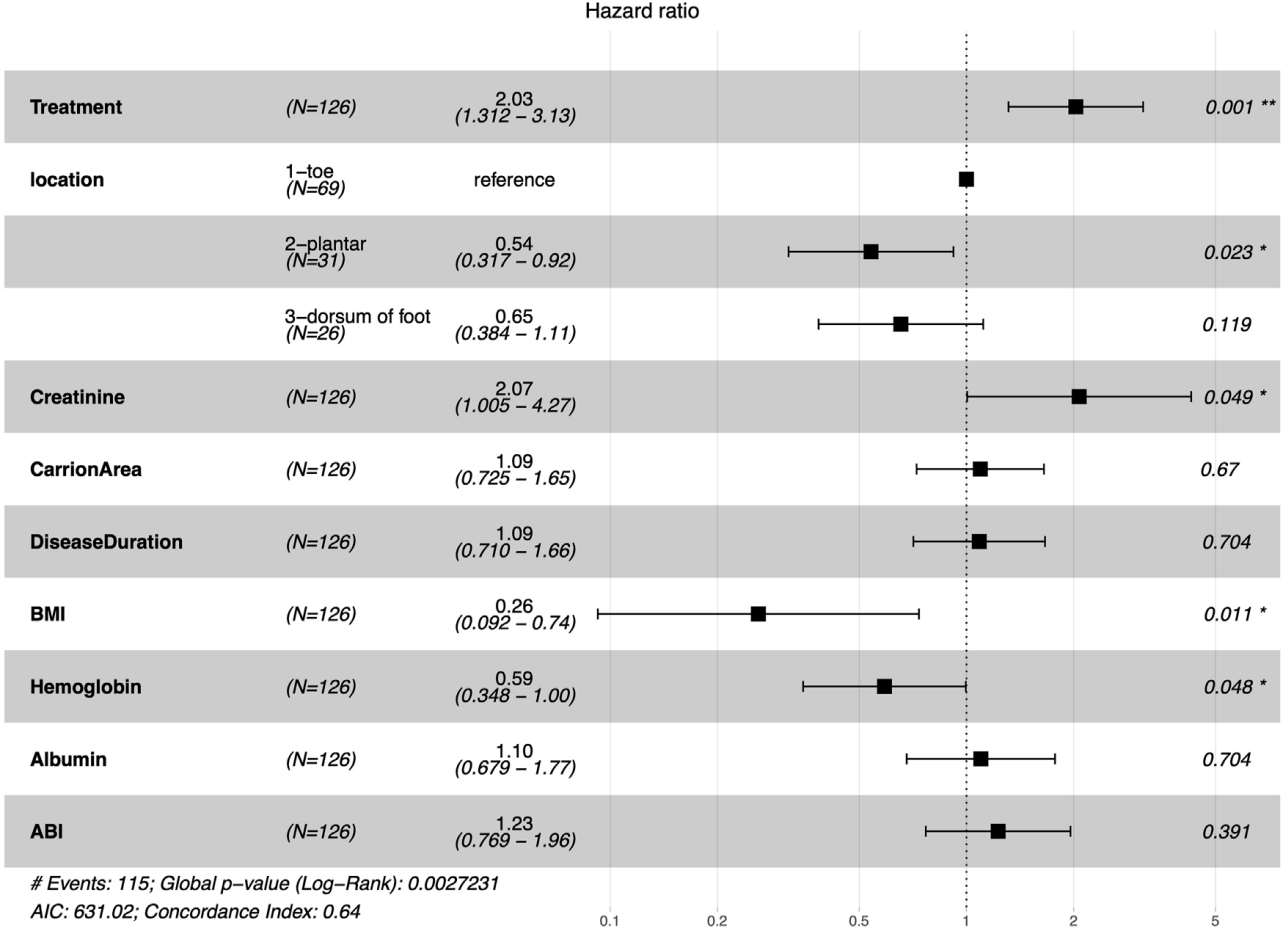
Results of the Cox proportional hazard model analysis. The forest plot depicted in this figure provides the Hazard Ratio (HR), the 95% Confidence Intervals (CIs), and the *p*-value of the HR for each selected covariate included in the Cox model.

**FIGURE 3:**
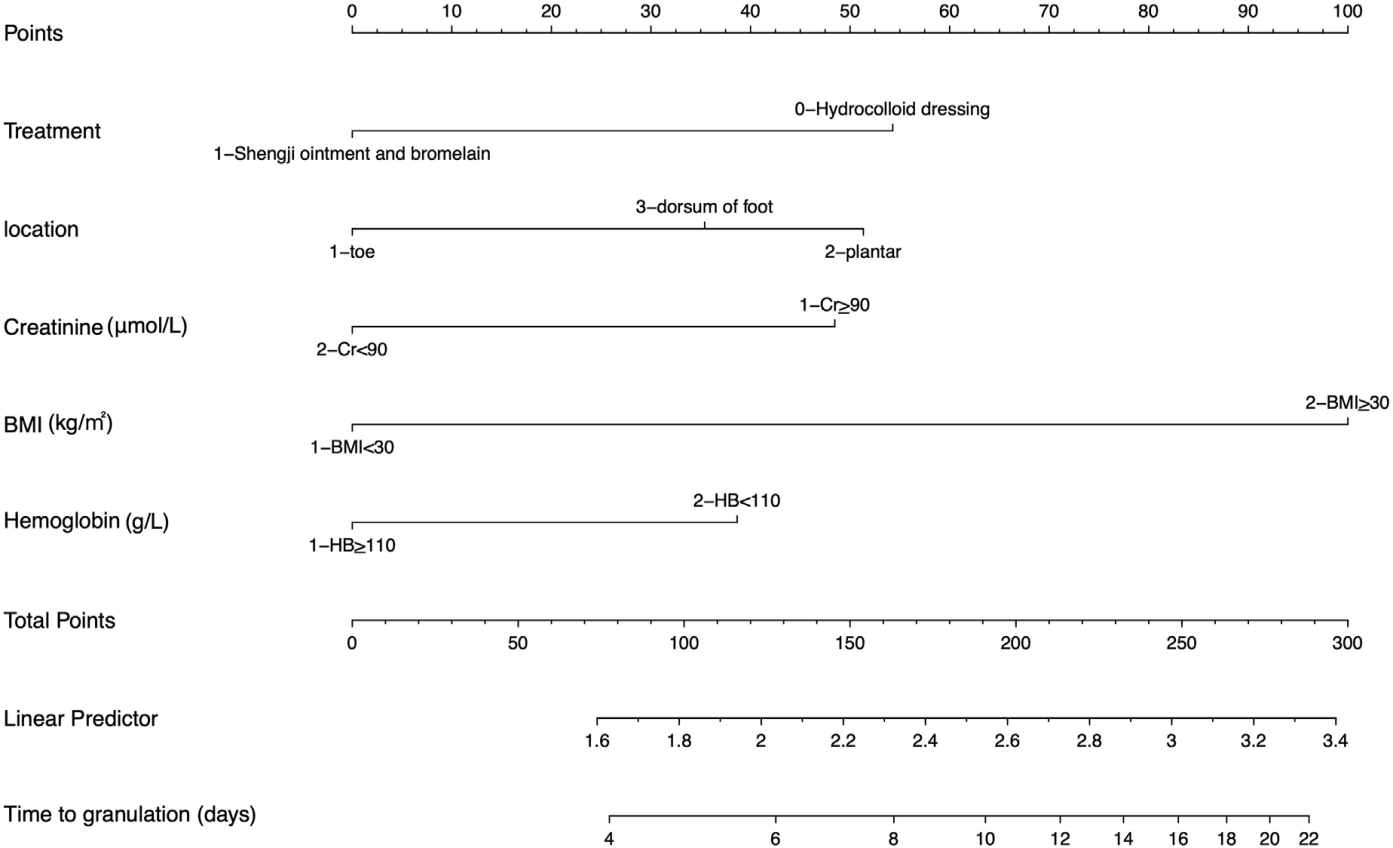
Nomogram of the prediction model. In the nomogram, each variable corresponds to a specific score. By summing up the scores for all variables, a total score can be calculated. This total score can then be used for the prediction of outcome variables.

A multi-factorial Cox proportional hazard regression model was constructed based on the independent variables identified via the lasso regression analysis. The time until granulation tissue formation served as the dependent variable, as depicted in Figure 2. The treatment approach, ulcer location, creatinine levels, Body Mass Index (BMI), and hemoglobin were identified as independent factors affecting the production of granulation tissue in diabetic foot ulcers. The combined treatment of Chinese herbal Shengji ointment and bromelain positively influenced granulation tissue development. Conversely, plantar ulcers, impaired renal function, obesity, and anemia were found to negatively impact granulation tissue formation. The results of the Cox proportional hazard model analysis are shown in Figure 2, and the predictive model nomogram is displayed in Figure 3.

### 3.4 Model evaluation

**FIGURE 4:**
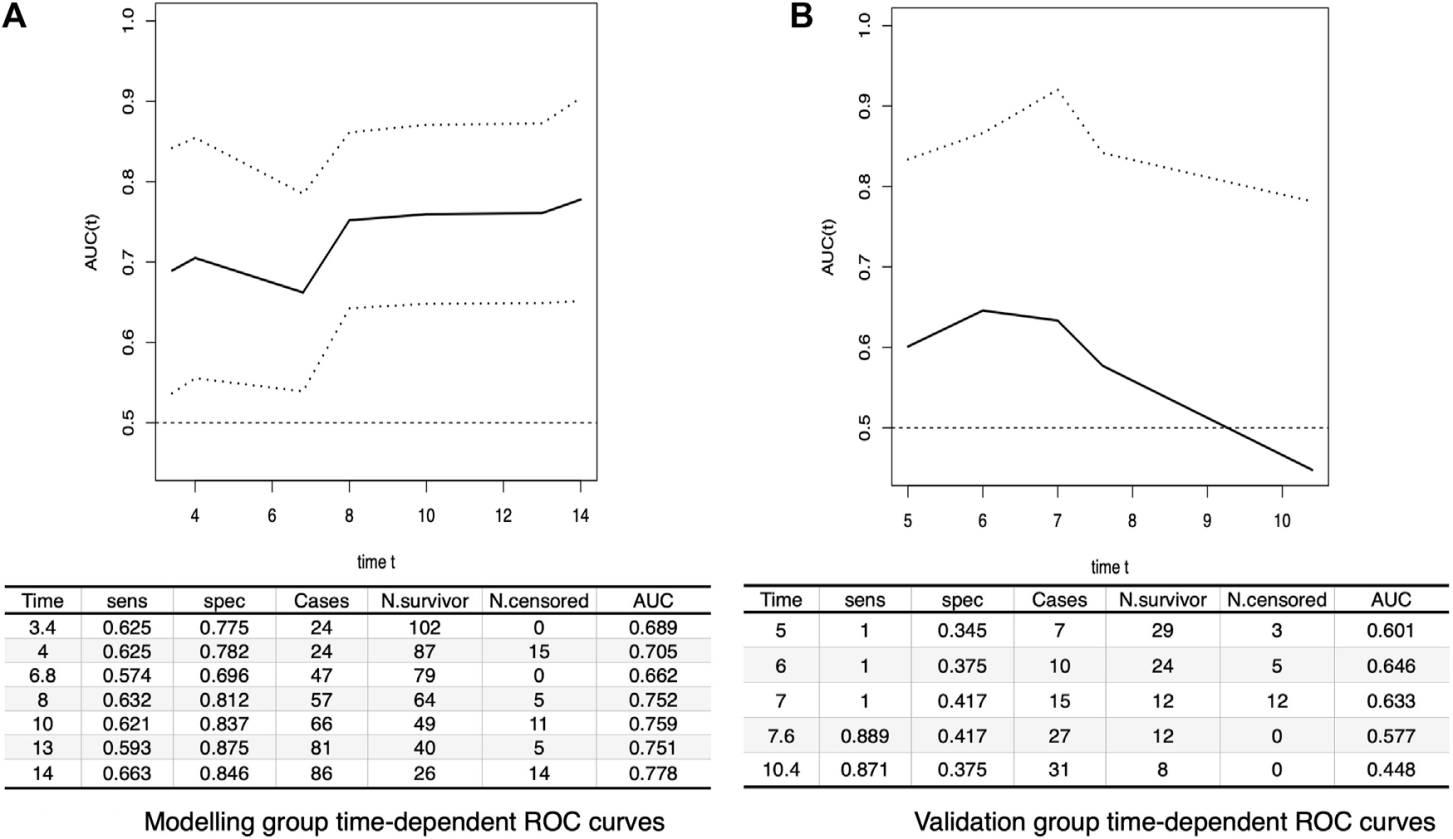
Time-Dependent ROC Curves. **(A)** This panel shows the results of the time-dependent ROC curve for the modeling group. The *x*-axis represents time, while the AUC and its 95% Confidence Intervals (CIs) are plotted on the *y*-axis. **(B)** This panel displays the results of the time-dependent ROC curve for the validation group. Similar to **(A)**, time is represented on the *x*-axis and the AUC along with its 95% CIs is plotted on the *y*-axis.

This study utilized the bootstrap method for both internal and external validation of the model. The accuracy of the model’s predictions was evaluated through time-dependent receiver operating characteristic curve analysis. The area under the curve (AUC) for the model group data set ranged between 0.7 and 0.8, demonstrating the model’s substantial predictive capability for the occurrence of granulation tissue in patients with diabetic foot ulcers post-treatment. These findings are depicted in Figure 4.

## 4 Discussion

### 4.1 Combined therapy with Chinese medicine may foster wound healing in diabetic foot ulcers (DFUs) of Wagner grade 3 or higher

Shengji ointment stimulates metabolism through a multitude of biochemical reactions such as hydrolysis, enzymatic digestion, acidification, saponification, and esterification, resulting in bactericidal and anti-inflammatory effects. It maintains a moderate level of immune response and controls the partial inflammatory response, a cornerstone for damage repair (Xu and Xiao, 2003). Moreover, it promotes the growth of fresh granulation and skin regeneration, maintains the trauma’s regenerative environment, encourages the contraction of the trauma, and facilitates epidermal cell differentiation, reproduction, migration, covering the trauma, ultimately leading to trauma healing (Ma and Ju, 2013).

Recent pharmacological studies propose its potential to mitigate inflammation by downregulating TNF-α and IL-6 proteins in wounded tissues (Sun et al., 2021). Furthermore, it may amplify the expression of VEGF protein within these tissues, fostering angiogenesis and enhancing microcirculation at the wound site, which subsequently aids wound healing (Song et al., 2020). The outcomes of some clinical trials imply that Shengji ointment exhibits excellent efficacy in skin ulcers that are hard to heal (Zhang, 2017; Xie, 2020). It has been found that the repair and regeneration of skin tissue damage correlate with the change in collagen content, and the type Ⅰ/type Ⅳ collagen ratio can effectively reflect the tissue repair in patients (Ye, 2018).

Shengji ointment can enhance the type Ⅰ/type Ⅳ collagen ratio and hasten collagen synthesis, thus promoting wound healing. The clinical manifestations include pus coverage on the wound surface, rapid growth of the wound edge, redness of the granulation tissue, easy bleeding when touched, and the emergence of “skin islands” in the center of the wound (Li et al., 2013). When applied externally to the ulcer surface, Shengji Ointment forms a protective film that isolates the wound surface from the external environment, reduces contamination, and possesses an anti-infective effect believed to be related to the activation of macrophages and enhancement of phagocytosis (Lu et al., 2018).

Meanwhile, bromelain, a protease derived from the pineapple plant, is another vital component in the treatment regimen, especially for DFUs exhibiting exposed tendon necrosis. Bromelain is extracted from the natural plant pineapple stem or unripe fruit, with the primary component being a large class of sulfhydryl protease, which includes phosphodiesterase, glycosidase, peroxidase, etc., and functions to hydrolyze proteins (Pereira et al., 2023). Bromelain works by breaking down collagen fibers, liquefying necrotic tendon and fascial tissues, and facilitating the removal of necrotic tissue while preserving the surrounding healthy skin, thereby creating an ideal environment for wound healing. Furthermore, it removes trauma-induced fibrin or clots, improves local circulation, reduces swelling and inflammation, and inhibits platelet aggregation, collectively accelerating blood flow and promoting wound healing (Ramundo and Gray, 2008; Zhang et al., 2012).

Our results have underscored the effectiveness of a combined treatment approach involving Traditional Chinese Medicine. The utilization of Chinese herbal Shengji ointment alongside bromelain appears to foster granulation tissue development, thereby expediting the healing process. This suggests that integrating Traditional Chinese Medicine into conventional treatment strategies for DFUs may provide enhanced therapeutic outcomes.

### 4.2 Ulcer location, hemoglobin, creatinine, and BMI are identified as independent risk factors for Wagner grade 3 or higher DFUs

Our study observed that plantar ulcers presented a greater healing challenge compared to toe and dorsum ulcers in patients with non-ischemic DFUs and controlled infection. Some research suggests that reducing plantar pressure may expedite DFU healing (Caravaggi et al., 2007); however, decompression therapy should not be solely relied upon for treating plantar ulcers but may be considered to reduce ulcer recurrence rates (Reiber et al., 2002).

Hemoglobin, acting as a nutritional status indicator, offers a partial reflection of a patient’s health condition. A national dietary survey exposed common misunderstandings about dietary management among diabetes patients and their families, such as believing in numerous food contraindications and advocating for extreme restrictions on consuming animal products, potentially leading to an insufficient intake of high-quality protein (Lin and Fan, 2006). When blood glucose is under robust control, dietary restrictions can be eased during the early stages of DFUs. As the disease progresses, patients should be encouraged to adopt a high-protein diet to aid in ulcer healing. In the late stages, when the ulcer fails to heal for an extended period and multiple debridements and dressing changes cause bleeding, active nutritional therapy emphasizing a high-protein diet and other nutritional intakes become necessary (Wei et al., 2015). Consequently, the clinical management of DFU patients should focus on personalized medical nutrition therapy, maintaining blood glucose levels within effective limits while strengthening nutritional support, particularly a high-protein diet. An appropriate dietary plan should be crafted for DFU patients at different stages, and dietary structure should be optimized to increase the protein intake proportion to facilitate DFU healing.

The study found that an increased BMI prolongs DFU healing time. Research also suggests that a high BMI is a significant risk factor for a diabetic foot, associated with increased foot pressure and adipokine accumulation-mediated vascular injury (Sohn et al., 2011). Furthermore, several studies indicate that BMI is linked with the development and prognosis of multisystem diabetes complications (Yang et al., 2017; Jia, 2020; Yang et al., 2020; Zhang et al., 2022), and suitable BMI control might improve the overall diabetes outcome.

High creatinine levels were identified as an independent risk factor for wound healing in diabetic foot patients without a history of chronic kidney disease. Research indicates that renal damage in diabetic foot patients becomes more severe as Wagner grade increases (Ding et al., 2015), possibly due to wound or recurrent infection-induced inflammatory factor release leading to renal damage (Game et al., 2013). This suggests that early multidisciplinary combination therapy should be administered to patients with Wagner grade 3 or higher to minimize the risk of progression to end-stage renal disease.

### 4.3 Limitations and future directions

While this study’s rigorous screening criteria—which excluded patients with Wagner grades 1–2 and those with co-infections, malnutrition, and poor lower extremity circulation—increased the internal validity by reducing confounding factors, it also limited the sample size. This strict selection process enhanced the study’s precision, but it also restricted the generalizability of the results.

Additionally, discrepancies in certain baseline variables (such as total bilirubin, indirect bilirubin, decay percentage, total wound area) were noticed during the external validation of the model, leading to a decrease in the area under the ROC curve for the externally validated model. This suggests that while the prediction model holds value, it still necessitates further refinement and validation using a larger and more diverse patient dataset. Moreover, the endpoint of this study is the formation of granulation tissue, and we hope that future research will focus on long-term survival indicators of patients.

Despite these limitations, the study provides valuable insights into the treatment of DFU, laying the groundwork for future research aimed at creating a more accurate clinical prediction model that could significantly enhance the prognosis and quality of life for patients with diabetic foot ulcers.

## 5 Conclusion

Through the application of Lasso regression and multi-factor Cox regression, this study successfully constructed a valuable predictive model. This model represents a potent tool for healthcare professionals to assess treatment efficacy and individualize treatment strategies for patients suffering from Wagner grade 3-4 diabetic foot ulcers. The ability to adapt and optimize treatment according to the specific needs and conditions of each patient is crucial in enhancing patient outcomes.

In this patient population, we discovered that the combined therapy of Chinese medicine ointment acted as an independent protective factor for wound healing. Conversely, plantar ulceration, reduced hemoglobin, elevated body mass index, and high creatinine levels were identified as independent risk factors. Collectively, these factors demonstrated predictive power for the outcomes of diabetic foot ulcers.

However, it is important to note that the findings of this study should be cautiously extrapolated as it excluded patients with severe infection or poor blood supply. These limitations underscore the need for more comprehensive and diverse samples to enhance the predictive model.

Despite these limitations, this study offers valuable insights into the treatment of deep diabetic foot ulcers in elderly patients with diabetes, underscoring the potential for integrating Chinese Medicine into treatment plans. With further validation and refinement, our predictive model could potentially be incorporated into routine clinical practice, contributing to improved patient outcomes.

## Data Availability

The raw data supporting the conclusion of this article will be made available by the authors, without undue reservation.
