# Supplementary material for: Predictive factors and clinical efficacy of Chinese medicine Shengji ointment in the treatment of diabetic foot ulcers in the elderly: a prospective study
Source: Front Pharmacol. 2023 Aug 16;14:1236229. doi: 10.3389/fphar.2023.1236229 (PMC10468590; doi:10.3389/fphar.2023.1236229)
Supplement: Supplementary file 1 [file Presentation1.pdf]

Standard operating procedure for wound debridement and dressing change

I. Debridement

(1) 0.9% saline from a 10-mL syringe is used to wash the ulcer before it is dried with sterile gauze.

(2) When necessary, debridement should be performed to remove necrotic tissue and exudate.

II. Dressing change

(1) 0.9% saline from a 10-mL syringe is used to wash the ulcer before it is dried with sterile gauze;

(2)

a) Intervention group (bromelain + Shengji ointment for external use): After debridement, bromelain is evenly spread on the wound surface before the Shengji ointment is applied to the wound surface in a 0.25-cm-thick layer. The ulcer is then wrapped with sterile gauze, bandaged, and fixated. The dressing is changed, and treatment is applied every 24 h.

b) Positive control group (hydrocolloid dressing): A sufficient amount of hydrocolloid dressing is used to cover the wound surface, ensuring that the dressing is at an even level with the skin around the wound. Sterile gauze is wrapped around the ulcer and fixated. The dressing is changed every 24 h.

III. Decompression

During the entire trial, patients will be advised to use appropriate

decompression, such as crutches, wheelchairs, orthoses, and decompression insoles. The method of decompression is recorded in the case report form throughout the trial.

Note: All debridement must be recorded in the case report form, and the daily dressing changes should be performed by trained researchers.
