# Supplementary material for: Predictive factors and clinical efficacy of Chinese medicine Shengji ointment in the treatment of diabetic foot ulcers in the elderly: a prospective study
Source: Front Pharmacol. 2023 Aug 16;14:1236229. doi: 10.3389/fphar.2023.1236229 (PMC10468590; doi:10.3389/fphar.2023.1236229)
Supplement: Supplementary file 3 [file Presentation3.pdf]

Identification of constituents in Shengji ointment

6 g Shengji ointment was heated, melted, and dispersed by diatomite evenly. 0.5 g drug-diatomite sample was added with 3 times methanol (0.1% formic acid in methanol), ultrasonically extracted for 20min, centrifuged for 10 min at 12000 r/min at 4℃. The supernatant was taken and centrifuged again for 10min under the same conditions.

2 μL of the sample supernatant injected into an Acquity UPLC HSS T3 column (2.1 mm × 100 mm, 1.8 μm, Waters, Milford, MA, USA), and was analyzed by the ultra-performance liquid chromatography with quadrupole time-of-flight mass spectrometry instrument (UPLC-QTOF-MS). The column temperature was set at 25 ℃, and the flow rate was 0.4 mL/min. The mobile phase consisted of solvent A (0.1% formic acid in water) and solvent B (0.1% formic acid in acetonitrile). The gradient for plasma was set as follows: 0−0.2 min, 85% A; 0.2−24 min, 85-0% A; 24−27 min, 0% A; 27−27.1 min, 85% A; 27.1−30 min, 85% A.

The MS data were acquired in the positive and negative ion modes using a data-independent acquisition approach (MS^E). An electrospray ionization source (ESI source) parameters were as follows: a capillary voltage of 3.0 kV for positive mode, of -2.5 kV for negative mode; cone voltage of 30 V; desolvation temperature of 400 ℃; desolvation gas flow of 800 L/h; source temperature of 120 ℃; ion scan, *m/z* 50-1500 Da.

Nine components of Shengji ointment were identified, as shown in Table S1. 3-N-butylphthalide and Ligustilide were identified as standard substances and characteristic fragments. Others were identified based on HMDB and the Massbank database in Fig. S1-S2.

Table S1. Components in Shengji ointment

Peak	Component	Formula	Time (min)	Ion type	Measured mass(m/z)	Error (ppm)	Fragments	Source	Reference
1	Azelaic acid	$C_9H_{16}O_4$	5.13	$[M-H]^-$	187.0967	2.67	125.0958, 126.0992, 123.0787, 97.0646	Angelica	Pubchem
2	N-butylidenephthalide	$C_{12}H_{12}O_2$	9.37	$[M+H]^+$	189.0916	0.53	171.0810, 161.0963, 147.0445	Angelica	Pubchem
3	3-N-butylphthalide	$C_{12}H_{14}O_2$	12.57	$[M+H]^+$	191.107	-0.52	173.0955, 145.1016, 135.0448	Angelica	Standard
4	Palmitoleic acid	$C_{16}H_{30}O_2$	13.23	$[M+H]^+$	255.2323	-0.31	237.2218, 219.2109, 185.1520	Angelica/ Beeswax	Pubchem
5	Ligustilide	$C_{12}H_{14}O_2$	13.78	$[M+H]^+$	191.1062	-5.23	173.1297, 163.1103, 155.1079, 149.0235, 145.1012	Angelica	Standard
6	Linolenic acid	$C_{18}H_{30}O_2$	15.82	$[M+H]^+$	279.2328	1.79	261.2218, 243.2102, 95.0855, 81.0702	Angelica/ Beeswax	Pubchem
7	Linoleic acid	$C_{18}H_{32}O_2$	18.81	$[M+H]^+$	281.2481	0.35	263.2373, 245.2272, 97.1012	Angelica/ Beeswax	Pubchem
8	Palmitic acid	$C_{16}H_{32}O_2$	21.11	$[M+H]^+$	257.2478	-0.89	239.2370, 221.2263	Angelica/ Beeswax	HMDB
9	Oleic acid	$C_{18}H_{34}O_2$	21.59	$[M+H]^+$	283.2636	0	265.2534, 247.2423	Angelica/ Beeswax	HMDB

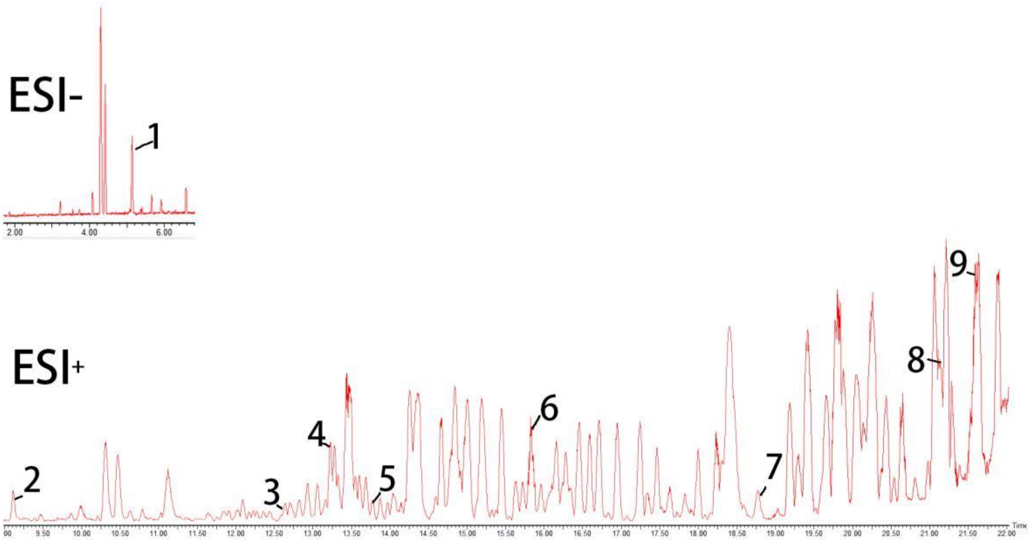

Fig. S1. UPLC-QTOF-MS chromatograms of Shengji ointment. Peaks: Azelaic acid (1), N-butylidenephthalide (2), 3-N-butylphthalide (3), Palmitoleic acid (4), Ligustilide (5), Linolenic acid (6), Linoleic acid (7), Palmitic acid (8), Oleic acid (9).

Fig. S2. MS/MS spectra of components in Shengji ointment.

1. Azelaic acid

PubChem

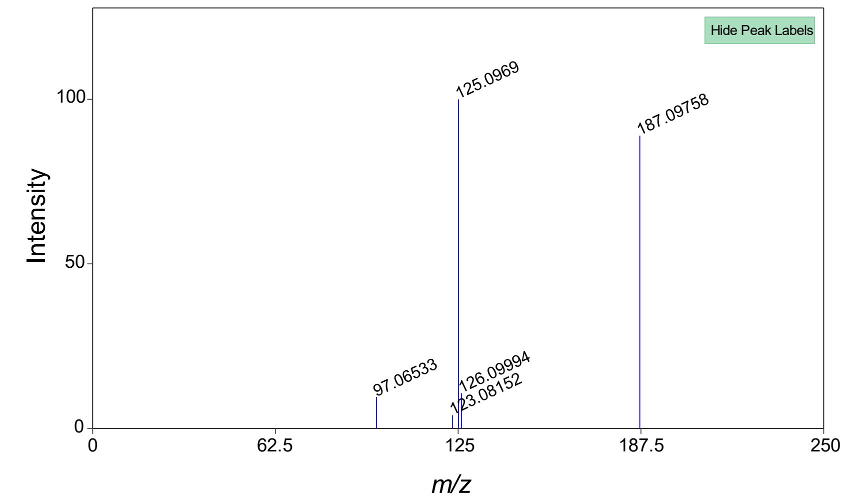

Sample

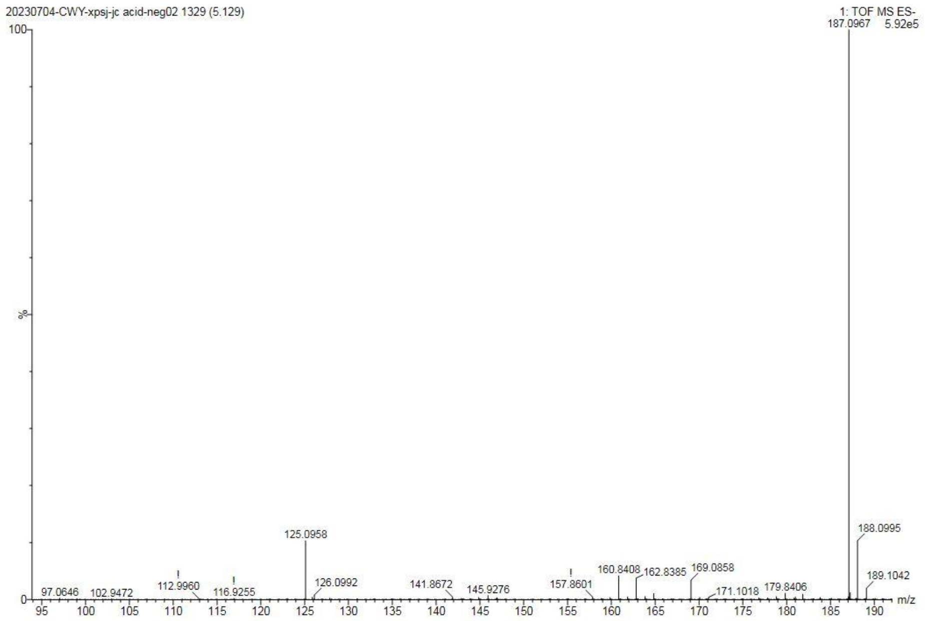

2. N-butylidenephthalide

PubChem

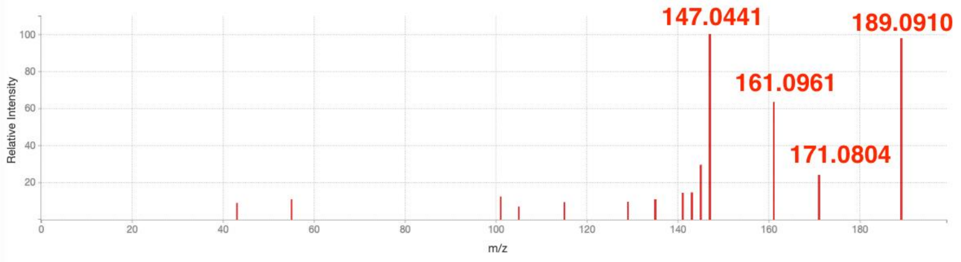

Sample	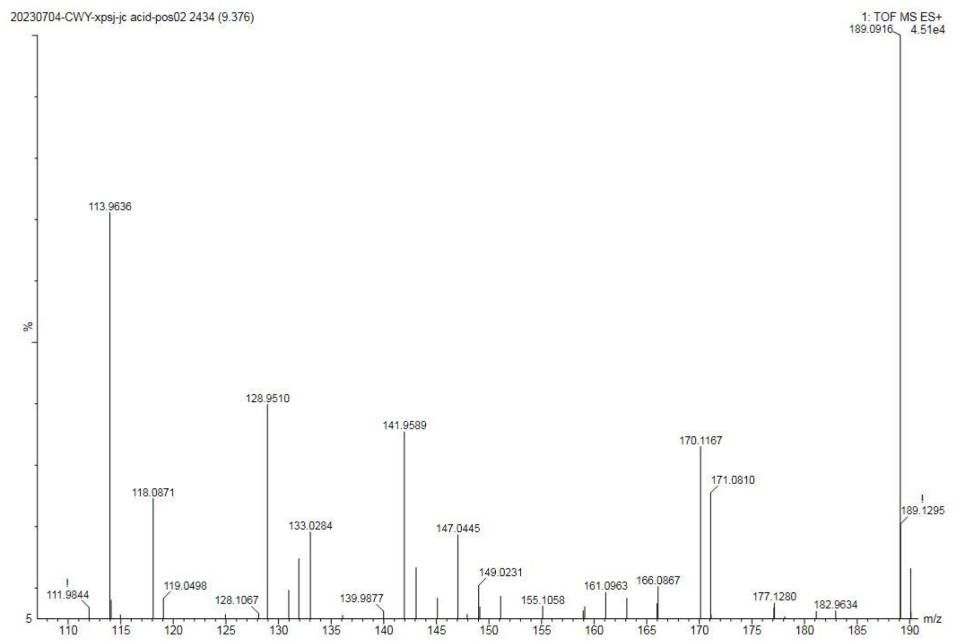

3. 3-N-butylphthalide

Standard	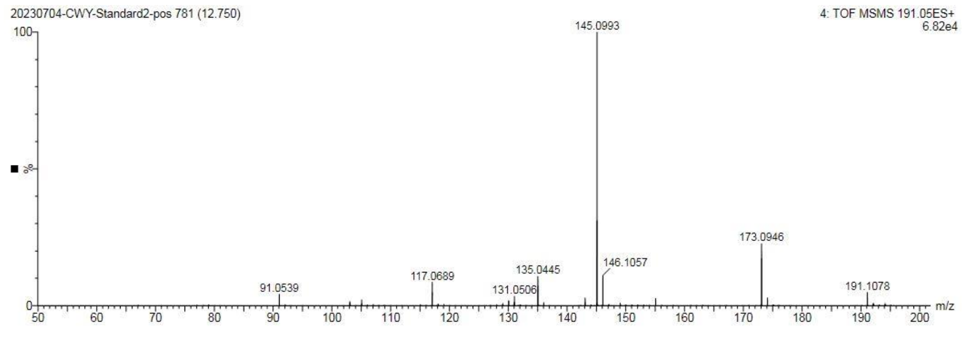
Sample	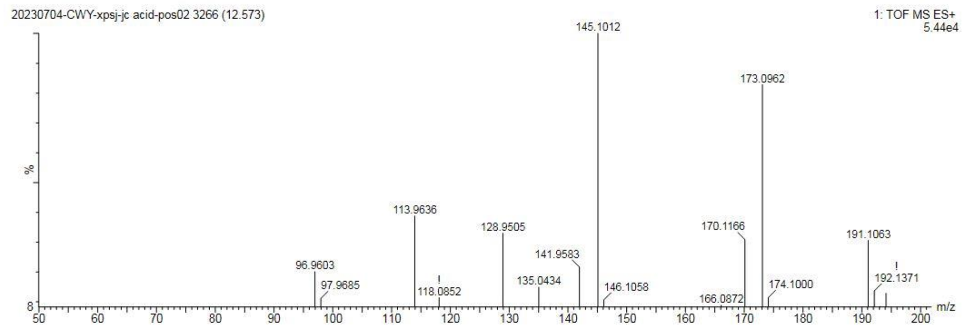

4. Palmitoleic acid

PubChem

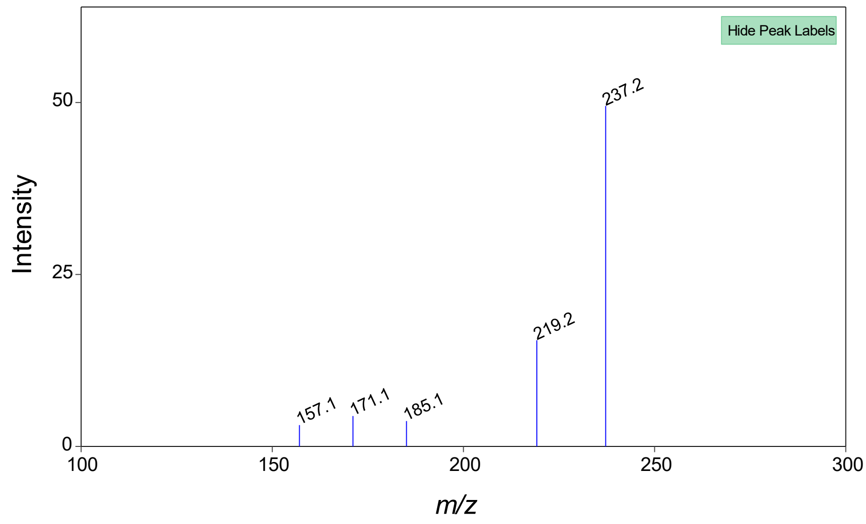

Sample

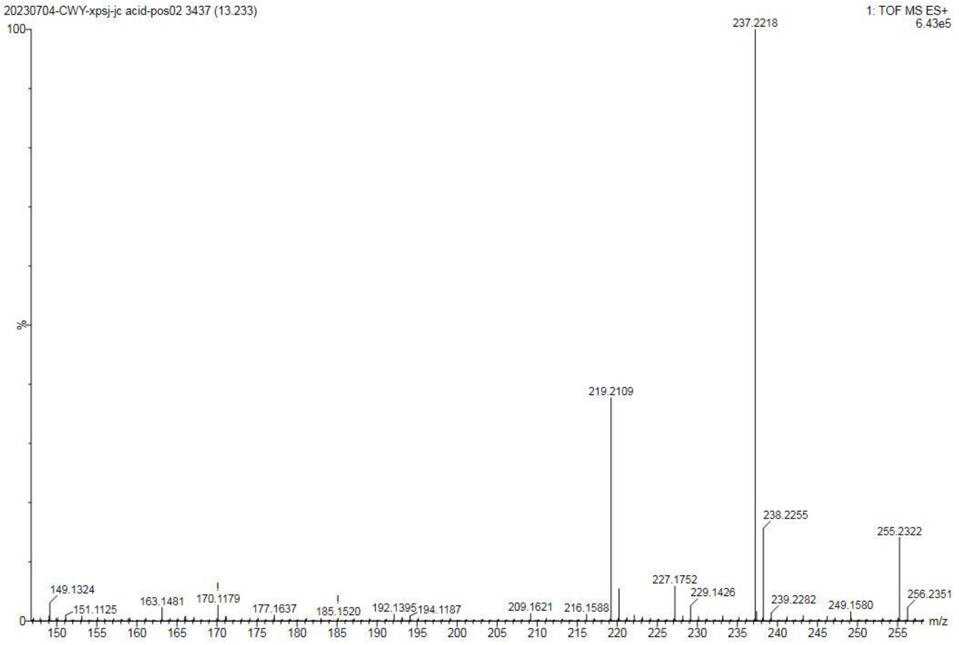

5. Ligustilide

Standard

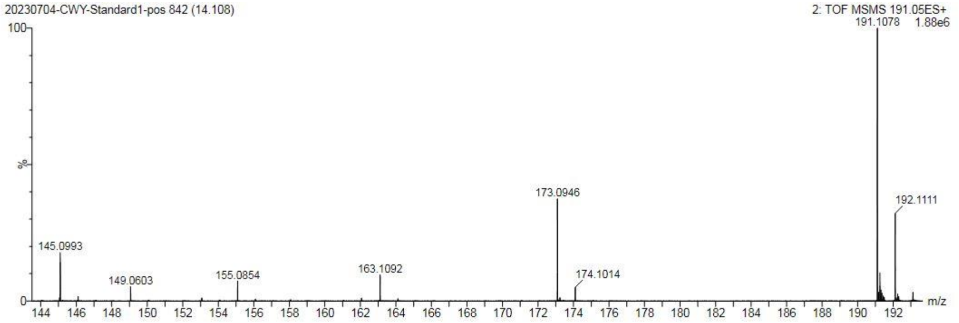

Sample	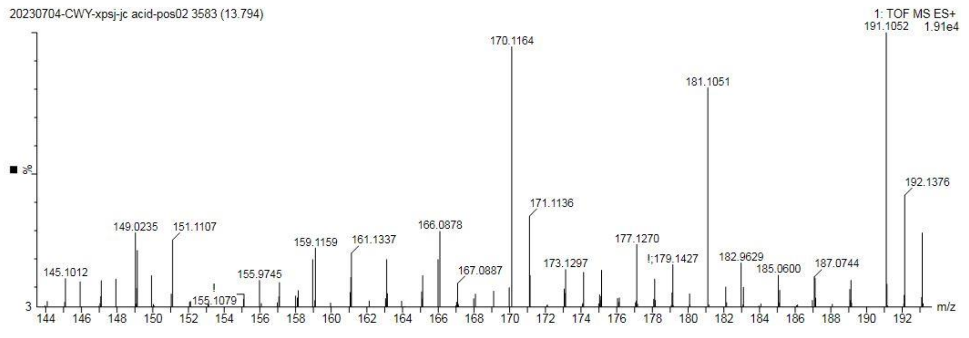

6. Linolenic acid

PubChem	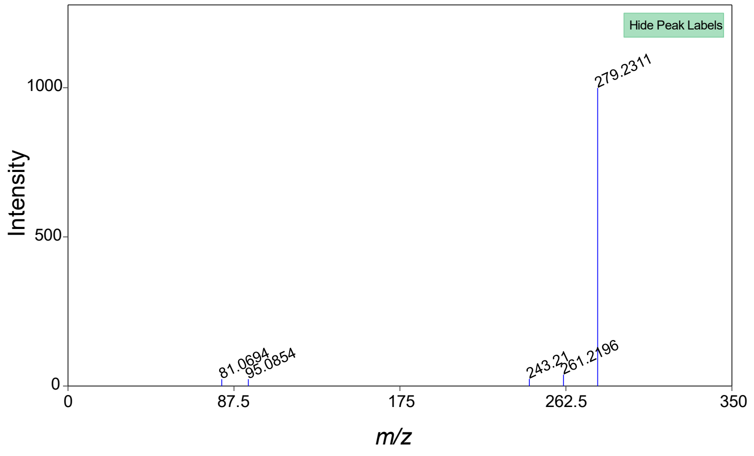
Sample	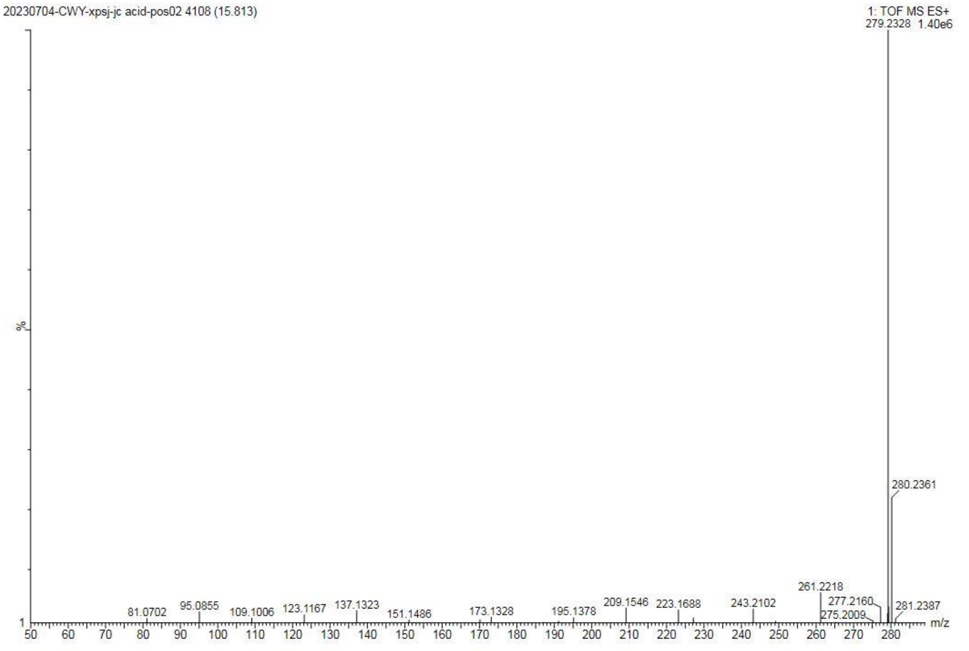

7. Linoleic acid

PubChem

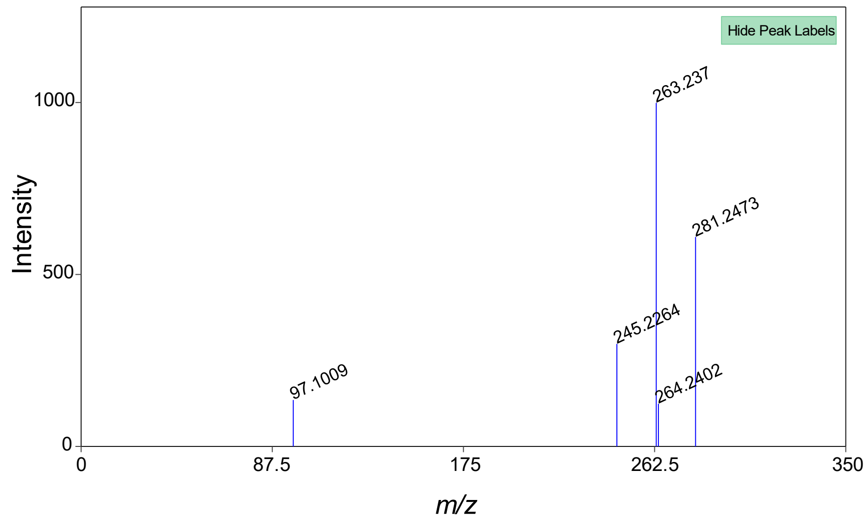

Sample

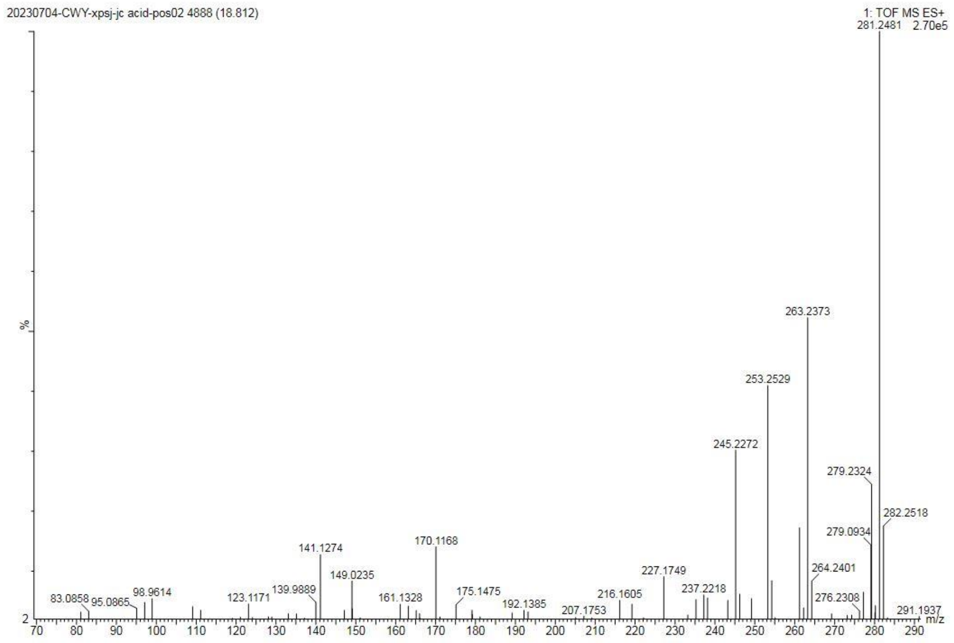

8. Palmitic acid

HMDB

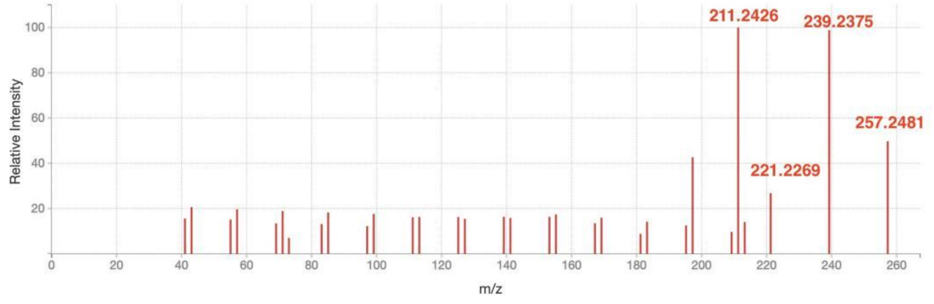

Sample	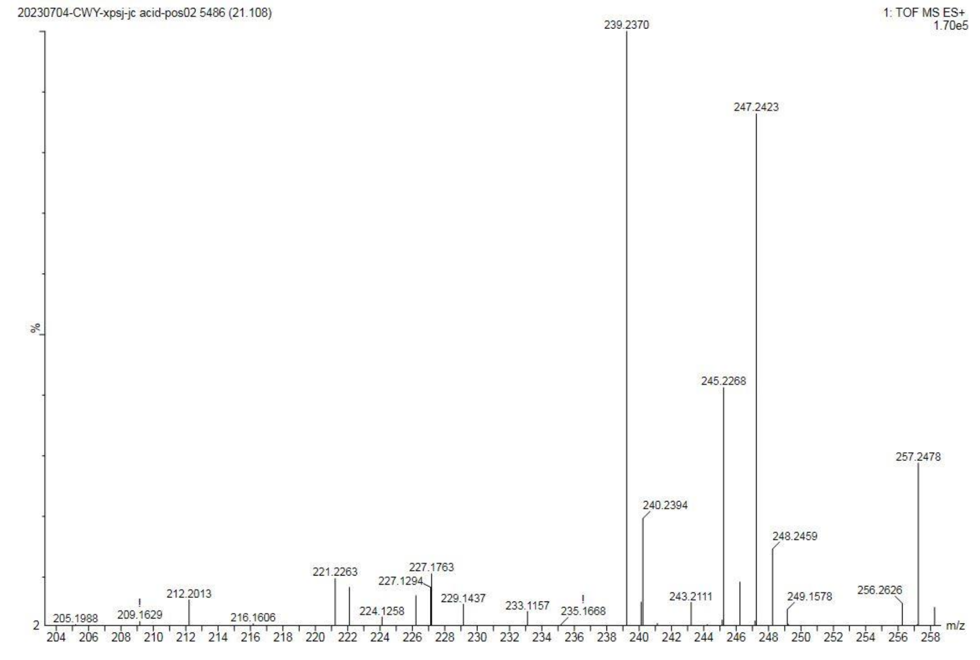

9. Oleic acid

HMDB	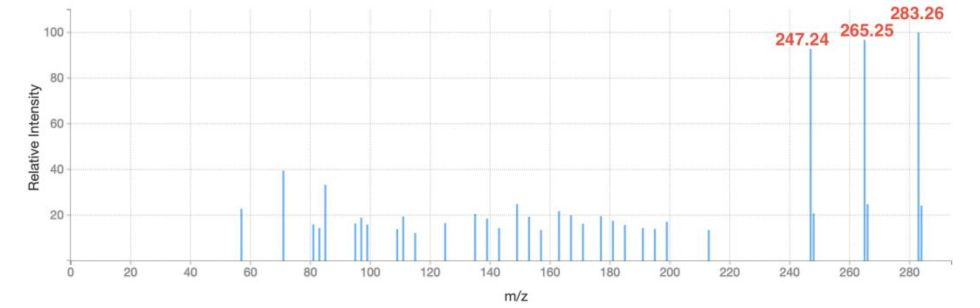
Sample	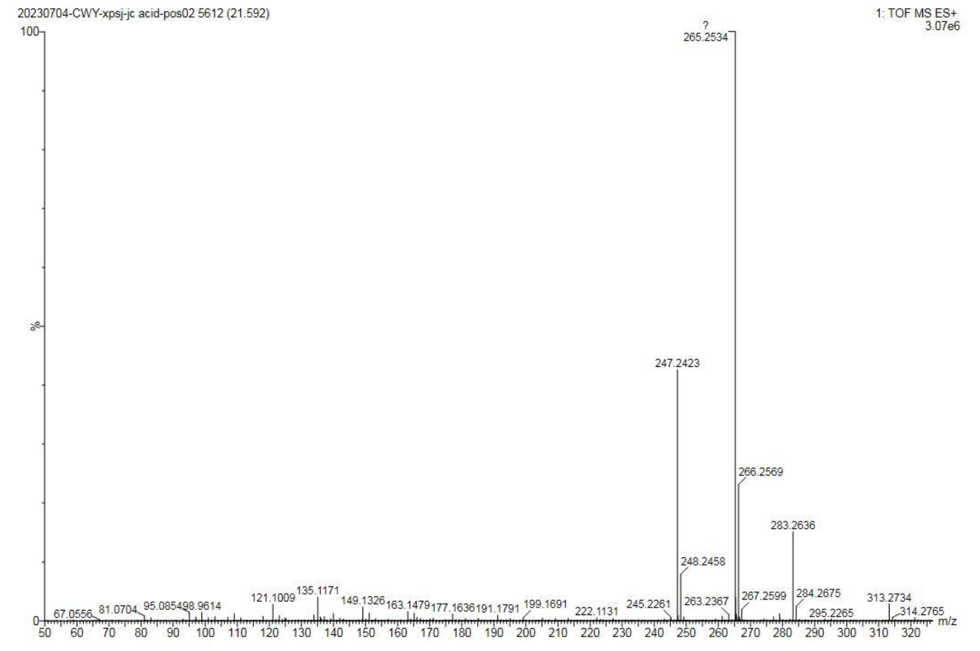

Formulation process of Shengji ointment

In the production process, Calamina and Gypsum are finely ground into powder and sieved for later use. Rehmannia glutinosa, Angelica sinensis and Crinis carbonisatus are roasted in a pot with sesame oil until dry, the residue is removed and the mixture is filtered. When it reaches a certain temperature, melted beeswax is added, followed by the addition of Calamina and Gypsum. The mixture is stirred evenly, cooled and then poured into containers to complete the process.

Quality control of Shengji ointment

Inspection Report of Tianjin Pharmaceutical Inspection and Research Institute			
**Test Name**	Shengji ointment	**Test ID**	YW202000285
**Manufacturer**	Tianjin Darentang Jingwanhong Pharmaceutical Co., Ltd, Tianjin, Tianjin, China	**Sample Provider**	Tianjin Darentang Jingwanhong Pharmaceutical Co., Ltd, Tianjin, Tianjin, China
**Expiry date**	2023-01-17	**Date of receipt of samples**	2020-03-24
**Packaging**	30g per bottle	**Dosage form**	Ointment
**Test item**	**Standard**	**Test Result**	
**Appearance**	Should be dark brown semi-solid ointment	Dark brown semi-solid ointment	
**Quantity**	Should be in accordance with the provisions	Compliance	
**Microbiological limit**	The total number of aerobic bacteria should not be more than 10^2cfu/ g	Less than 10cfu/ g	
	The total number of moulds and yeasts shall not exceed 10 cfu / g	Less than 5cfu/ g	
	Staphylococcus aureus should not be detected	Not detected	
	Pseudomonas aeruginosa should not be detected	Not detected	
**Test Conclusion**	This product was tested for the above items in accordance with National Medical Products Administration (NMPA) National Drug Standard Revision Approval(2001ZFB0067) and National Medical Products Administration (NMPA) Supplementary Drug Application Approval(2017B02035)and the results were in accordance with the regulations. 2020-6-8.		
